# Pathways and enhanced evaluation system for green low-carbon development across diverse Chinese regions

**DOI:** 10.3389/fpubh.2024.1465896

**Published:** 2024-11-25

**Authors:** Wenjie Li, Dechao Hu, Zongqi Xu, Jie Guo, Jianan Liu, Yuan Zhou

**Affiliations:** ^1^The Academy of Social Sciences on Water Research in the New Era, North China University of Water Resources and Electric Power, Zhengzhou, China; ^2^School of Management and Economics, North China University of Water Resources and Electric Power, Zhengzhou, China; ^3^Institute of Social Governance Research Center, Henan University, Kaifeng, China; ^4^School of Public Administration, North China University of Water Resources and Electric Power, Zhengzhou, China

**Keywords:** low carbon development, economic transition, green economy, fsQCA, sustainable development

## Abstract

**Background:**

Rapid economic growth in China has led to significant resource and environmental challenges, particularly in less economically developed regions. This study aims to identify effective strategies for achieving green, low-carbon development in these regions during the economic transition.

**Methods:**

We employed the Fuzzy-Set Qualitative Comparative Analysis (fsQCA) method to scrutinize the impact of economic, demographic, industrial, and technological factors on low-carbon development across a selection of Chinese provinces, including Qinghai, Hunan, Beijing, Shanghai, Tianjin, Hainan, and Chongqing. This approach facilitates a nuanced exploration of the multifaceted determinants of low-carbon progress within the regional contexts of China.

**Results:**

The study identified three distinct paths to low-carbon development, each with unique prioritization characteristics. These paths are the traditional early low-carbon path, the semi-modernized mid-carbon path, and the post-modernized low-carbon path. Each path offers tailored strategies for less developed regions to enhance their environmental innovation capacity and global competitiveness.

**Conclusion:**

This research contributes a novel perspective for regional sustainable development in China by offering tailored low-carbon development strategies for less developed regions. The findings suggest that region-specific strategies, aligned with developmental stages and characteristics, are essential for ensuring balanced economic, social, and environmental development.

## 1 Introduction

**Figure 1:**
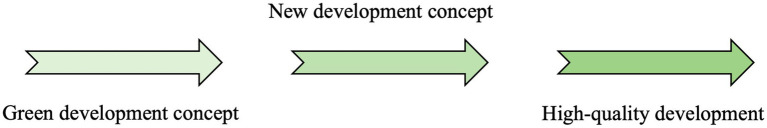
Related concepts of green development in China.

Sustainable development discussions often center on the interplay between economic growth and sustainability initiatives (1, 2). China’s rapid industrialization presents ecological and resource challenges, prompting a move toward sustainable, low-carbon industries that balance economic advancement with environmental conservation (3, 4). Figure 1 illustrates China’s unique strategy, which integrates production and lifestyle changes for a harmonious economic and environmental model (5).

**Figure 2:**
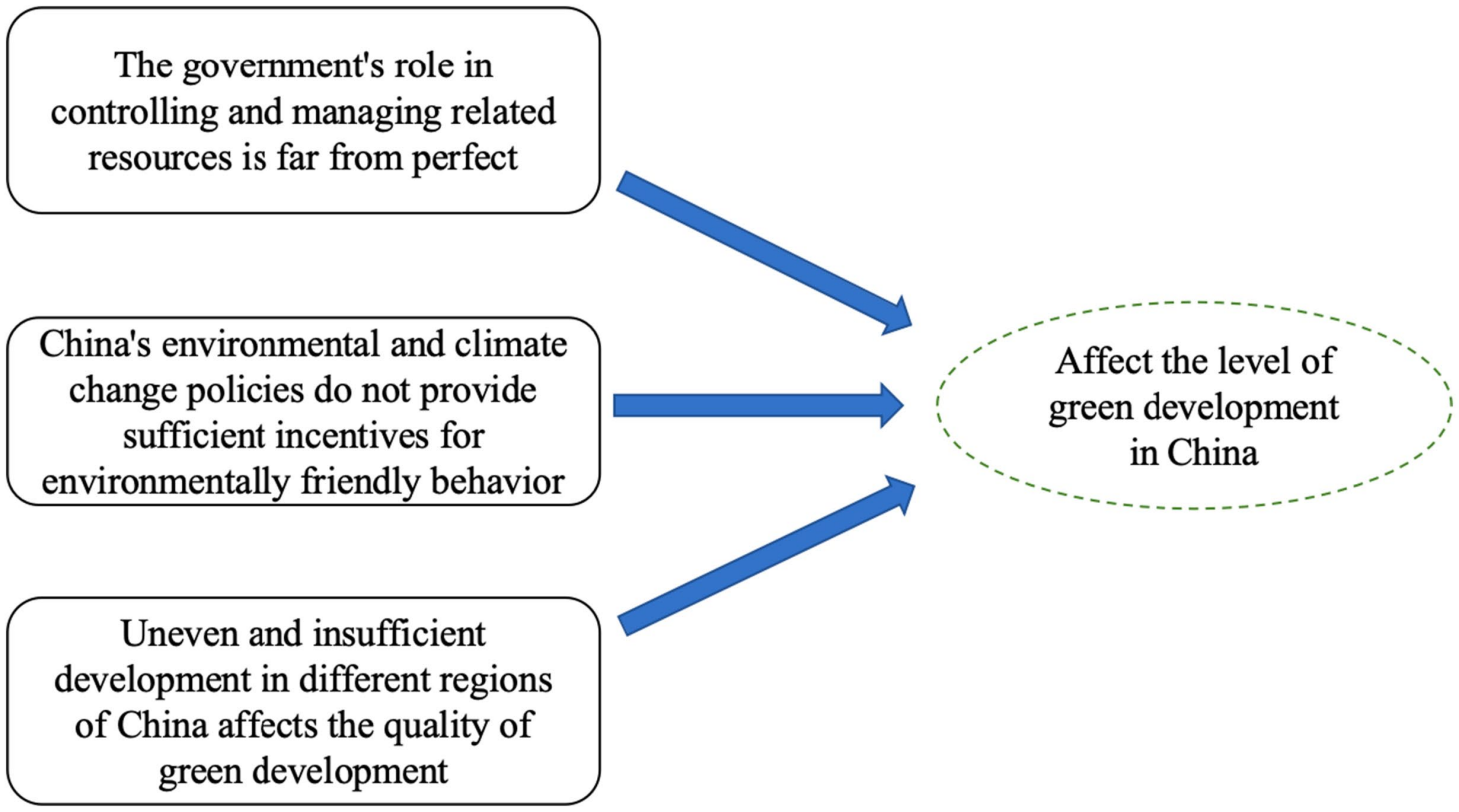
Negative factors affecting the level of green development in China.

Globally, the imperative for green and low-carbon development is acknowledged in addressing environmental and resource challenges (6). In China, swift economic expansion has led to pollution and degradation, especially in specific regions (7), prompting environmental protection measures (8). However, ongoing challenges are posed by the evolving nature of government interventions and insufficient incentives within current policies, which impact the effectiveness of green development (Figure 2) (9). Moreover, regional disparities are impeding equitable progress in green initiatives, influencing social inequality and welfare as the country moves toward high-quality development.

Figure 2 encapsulates the multifaceted factors influencing China’s pursuit of green development, exploring the interplay between micro-level practices and macro-level strategies. At the micro-level, businesses and communities are directly affected by environmental regulations and climate policies, which shape their green initiatives and operational efficiency. Meanwhile, at the macro-level, these factors are integrated into China’s economic structure and development plans, including government-driven green policies that foster the adoption of clean energy and low-carbon technologies. The figure also highlights the need to manage regional disparities and achieve a harmonious balance between economic advancement and environmental sustainability, indicating a complex, interconnected system of factors at work.

In fact, the Chinese economy has been growing at a very high rate. As a result, China has become the second largest economy in the world. Nevertheless, this rapid growth has caused a number of environmental problems, including pollution of the natural environment and depletion of natural resources (10). This is not only a devastating economic blow to the balance of natural resources and ecosystems, but also a serious threat to the health of all mankind. Despite China’s steady economic growth, its ability to innovate has not kept pace. This means that China’s economic development faces serious environmental problems, including pollution, energy shortages and ecological imbalances (11). This can affect the quality and healthy development of China’s society and economy to some extent. As a result, relevant authorities should implement environmental protection measures and make reasonable use of fiscal and financial measures to effectively address environmental issues (11). In addition, a series of policy measures are needed to better direct the flow of funds to environmental protection and other environment-friendly industries, and to reduce the allocation of funds to highly polluting industries (12). In conclusion, in recent years, China has been paying more and more attention to green and low-carbon development.

At present, China’s economy is developing rapidly and its economic power has risen to the second in the world. However, the economic development of the developed eastern coastal regions and the less developed central and western regions has become unbalanced and insufficient (13). As a result, there is a need to balance the economic development of the various regions of China. Strengthening the economies of the less developed regions is an effective and fast way to achieve this goal. After all, the less developed regions of the economy have always been a weak link in China’s economic development (14). In addition, the number of provinces in less economically developed regions accounts for a large portion of China. Indeed, if the economic performance of the less economically developed regions improves, they will contribute significantly to the economic development of China as a whole. A green economy will not only bring changes to the less economically developed regions, it will also increase the environmental innovation capacity of these regions (15). This will enable the less economically developed regions to improve their global competitiveness in terms of development. As such, the development of green economy in less economically developed regions needs to be studied in more detail.

To unlock greater growth potential for the green economy, robust support from green finance is essential. Simultaneously, establishing a robust and regulated green financial system is imperative to elevate the costs associated with pollutant emissions (16). This necessitates encouraging companies to transition from conventional innovation to green industries, such as high-tech and environmental protection. Crucially, financing green objectives can be facilitated by steering consumers toward environmentally friendly concepts, including environmental protection and green consumption (17). However, at its core, green financing encounters an inherent conflict with the objective of balancing environmental protection and sustainable economic development. Traditional economic development has, in many cases, contributed to environmental damage, and prioritizing environmental protection may require forgoing certain aspects of economic growth (18). Therefore, achieving green economic development in China requires the establishment of a robust green fiscal policy system for the less economically developed regions, which is pivotal from both theoretical and practical perspectives (19).

**Figure 3:**
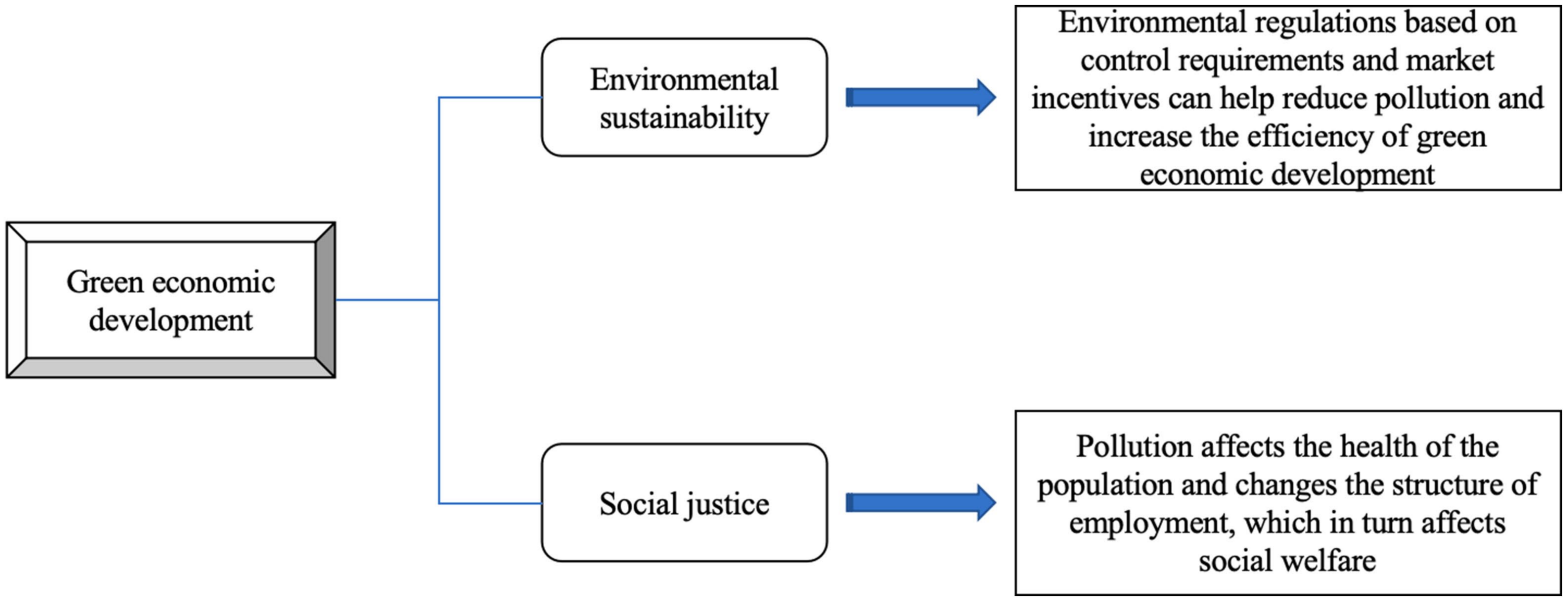
Aspects of green economic development.

In modern societies, pollution is a major barrier to green growth. In China, early economic development was based on a consumption-and emission-intensive industrial model. This meant that pollution problems were widespread in China and resources and the environment were in a desperate state (20). This situation has not only taken a heavy toll on the economy, but also poses a major threat to public health. The appropriate use of green, low-carbon technologies can contribute significantly to the growth of a green economy (21). More specifically, there are two aspects of green economic development: environmental sustainability and social justice (Figure 3). From the perspective of environmental sustainability, environmental regulations based on control requirements and market incentives can help reduce pollution and increase the efficiency of green economic development, thus promoting green economic development. From a social justice perspective, pollution affects the health of the population and alters the employment structure, consequently impacting social welfare (22). However, green low-carbon technologies can help reduce social inequality and improve social welfare (23). Consequently, it is imperative for researchers and policymakers to address pollution issues in China’s less economically developed regions by utilizing green and low-carbon technologies. This approach is crucial for steering the country’s economy toward a more sustainable and inclusive direction (24).

A comprehensive and unbiased scientific analysis of the green economy’s development necessitates a thorough understanding of the prevailing circumstances and influential factors (25). In essence, there is an escalating demand for evaluating and scrutinizing the green economy. Presently, numerous Chinese scholars and institutions are actively seeking objective methodologies to assess the green economy (26). While some research has explored various aspects of green economic development in these regions, a thorough and systematic assessment of the overall progress and the economic benefits derived from green and low-carbon initiatives is still limited.

Employing the fsQCA methodology, this study investigates the interplay of economic, demographic, industrial, and technological factors. It identifies and evaluates three distinct development paths, each offering targeted strategies that emphasize a harmonious balance between economic growth and environmental sustainability. The study’s findings suggest that by adopting these tailored strategies, less developed regions can enhance their environmental innovation capacity and global competitiveness, significantly contributing to China’s overall economic progress and ecological enhancement. Furthermore, the research underscores the pivotal role of green finance and the establishment of a robust green fiscal policy system in facilitating the transition to a low-carbon economy. This work contributes to the field by providing a nuanced understanding of regional sustainable development in the context of China’s economic transition, offering practical insights for policy formulation and strategic planning in less developed areas.

The contribution points of this study are as follows:

1. The research employs the Fuzzy-Set Qualitative Comparative Analysis (fsQCA) method to identify three distinct pathways for low-carbon development, each with unique prioritization characteristics, offering a nuanced understanding of how different regions can approach sustainable growth.
2. The study investigates the complex causal relationships between economic, demographic, industrial, and technological factors, providing a holistic view of the driving mechanisms of low-carbon development.

This research is organized as follows: Section 1 highlights the imperative shift toward green, low-carbon development in China amidst economic growth and environmental challenges. Section 2 outlines China’s strategy for green development, emphasizing the integration of environmental sustainability into economic growth and the critical role of green finance in supporting less developed regions. Section 3 outlines the fsQCA method to analyze factors influencing low-carbon development and the model construction for this research. Section 4 compares empirical results of different low-carbon development paths and their impact on regional sustainability. The final section concludes the importance of tailored low-carbon strategies and green finance for China’s less developed regions, suggesting directions for future studies.

## 2 Green low-carbon development in less developed regions

### 2.1 Green development theory

**Figure 4:**
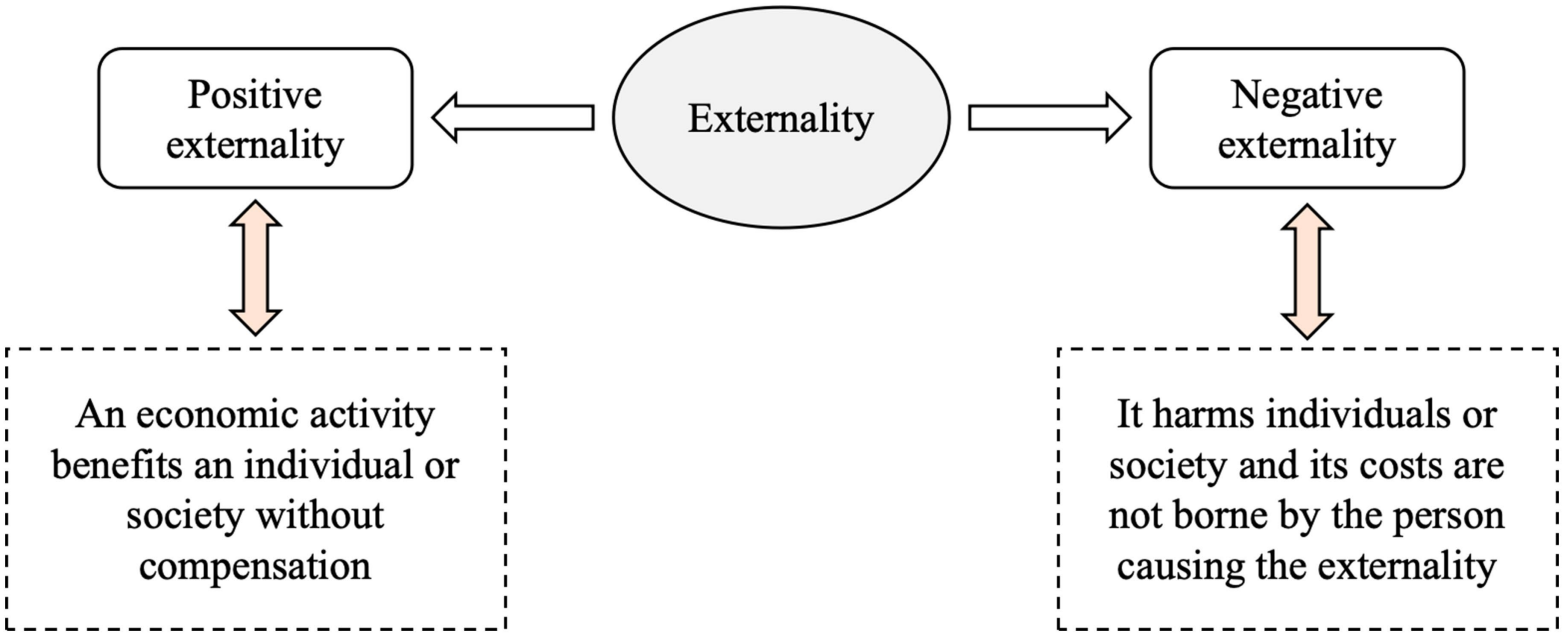
Positive externality and negative externality.

The theory of green development is derived from externality theory (27). The theory of green development is fundamentally rooted in the concept of externalities, which are the unintended side effects of economic activities that affect third parties who are not directly involved in the transaction. According to externality theory, these effects can be either positive or negative. Positive externalities occur when an economic activity benefits individuals or society without compensation, while negative externalities arise when the activity imposes costs on individuals or society that are not borne by the entity causing the externality. Sustainable economic growth can only be achieved if policies are designed to correct market failures associated with externalities. This can involve implementing taxes or regulations to discourage negative externalities, such as carbon taxes to reduce greenhouse gas emissions, or providing incentives for activities that generate positive externalities, such as subsidies for renewable energy projects. By addressing both the costs and benefits that are not reflected in market prices, green development theory aims to create a more accurate and equitable economic system that promotes long-term sustainability and environmental stewardship. Figure 4 provides a visual representation of the concept of externalities, delineating both positive and negative manifestations. Specifically, positive externalities are depicted as instances where economic activities confer unanticipated benefits upon individuals or the broader society without direct compensation. Conversely, negative externalities are portrayed as scenarios where such activities impose unintended costs on third parties or the environment, again, without appropriate compensation mechanisms in place. This dichotomy underscores the complexity of externalities and the necessity for a nuanced approach in economic policy and decision-making.

China has charted a distinctive course tailored to its national circumstances in pursuing the path of green development. More precisely, pertinent government departments have advocated for green and efficient production and lifestyle models, exemplified by the green development concept, the new development concept, and high-quality development. In essence, China’s green development concept underscores the metamorphosis of production methods and lifestyles (28). Explicitly, the Chinese government aspires to foster a model of economic growth where economic development and environmental protection synergize and complement each other. This model is inherently characterized by a low-input, high-output, low-pollution, and high-efficiency approach to both production and living.

**Figure 5:**
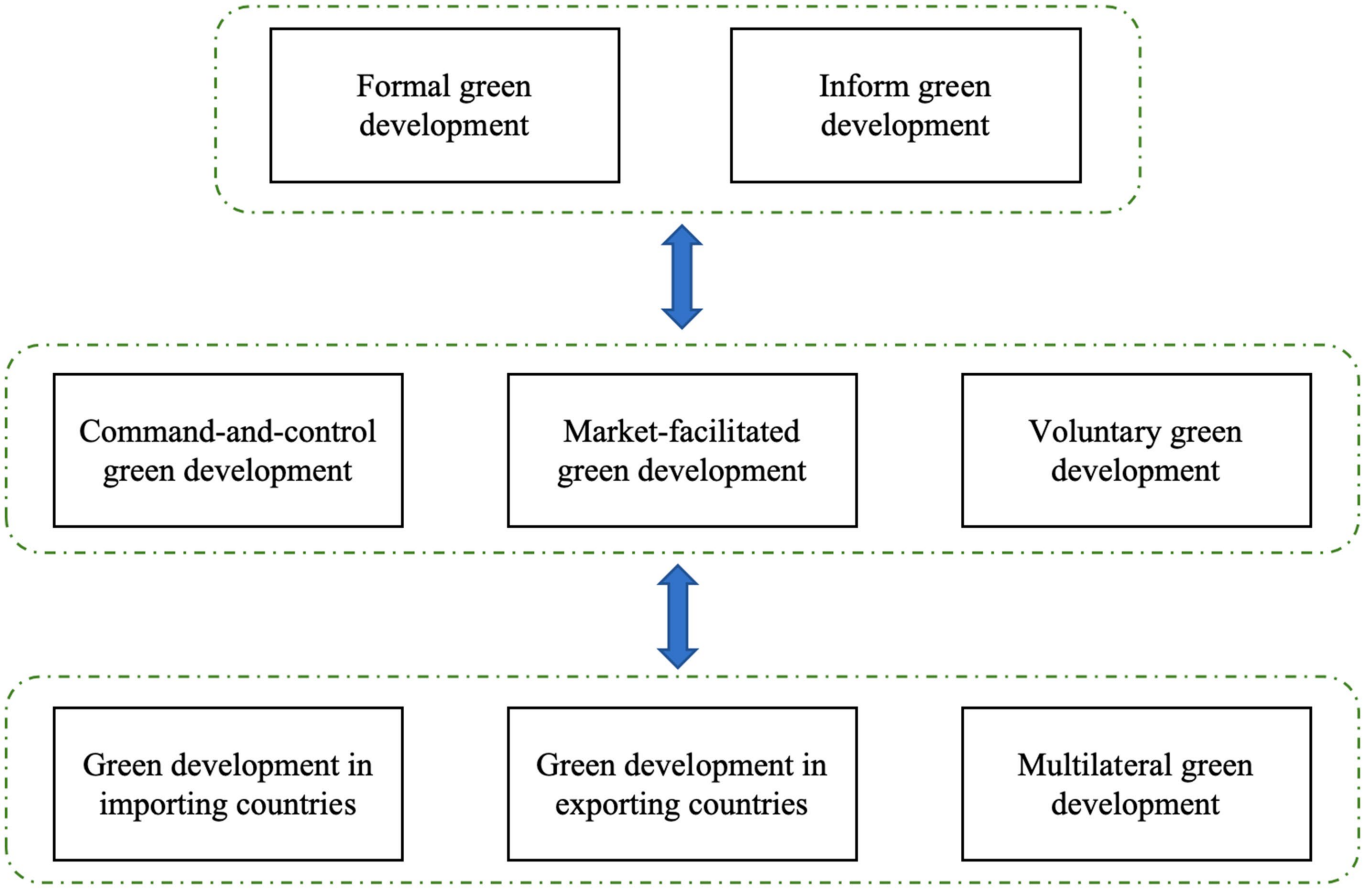
Classification of green development.

A reasonable classification of green development is a prerequisite for giving full play to its low-carbon impact. According to different classification criteria, green development can be classified in different ways (Figure 5). First and foremost, green development can be classified into formal as well as informal green development (29). In practice, this classification is the most commonly used. Formal green development refers to policies, measures and legal agreements issued by governments and administrative authorities to different economic actors. Informal green development, on the other hand, is based on the initiative of the participants. As a result, it is complementary to governmental green development. Secondly, according to the participants of green development, it can be divided into command-and-control green development (30), market-facilitated green development (31), and voluntary green development (32). What is more, according to the scope of the role of green development, it can be divided into green development in importing countries, green development in exporting countries, as well as multilateral green development.

### 2.2 Advancing the green economy: unveiling financial mechanisms for sustainable development

From an economic standpoint, the environment exhibits significant externalities, implying that economic agents neither receive benefits nor bear responsibility for safeguarding or polluting the environment. Green finance, in essence, seeks to reconcile environmental considerations with economic activities. Consequently, green finance also entails externalities.

In the bygone era of the planned economy system, China witnessed robust economic dynamism; however, this development model gave rise to profound environmental challenges. For instance, companies, in their pursuit of profit maximization, often neglected their social responsibilities. Moreover, many banks prioritized short-term gains and lacked a green business philosophy.

Conversely, the green economy encompasses a broader spectrum of economic activities and policies aimed at fostering sustainable development, emphasizing resource efficiency, and minimizing environmental impacts. The theory of green development, derived from externality theory, forms the foundation for the green economy, acknowledging the positive and negative externalities associated with economic activities.

**Figure 6:**
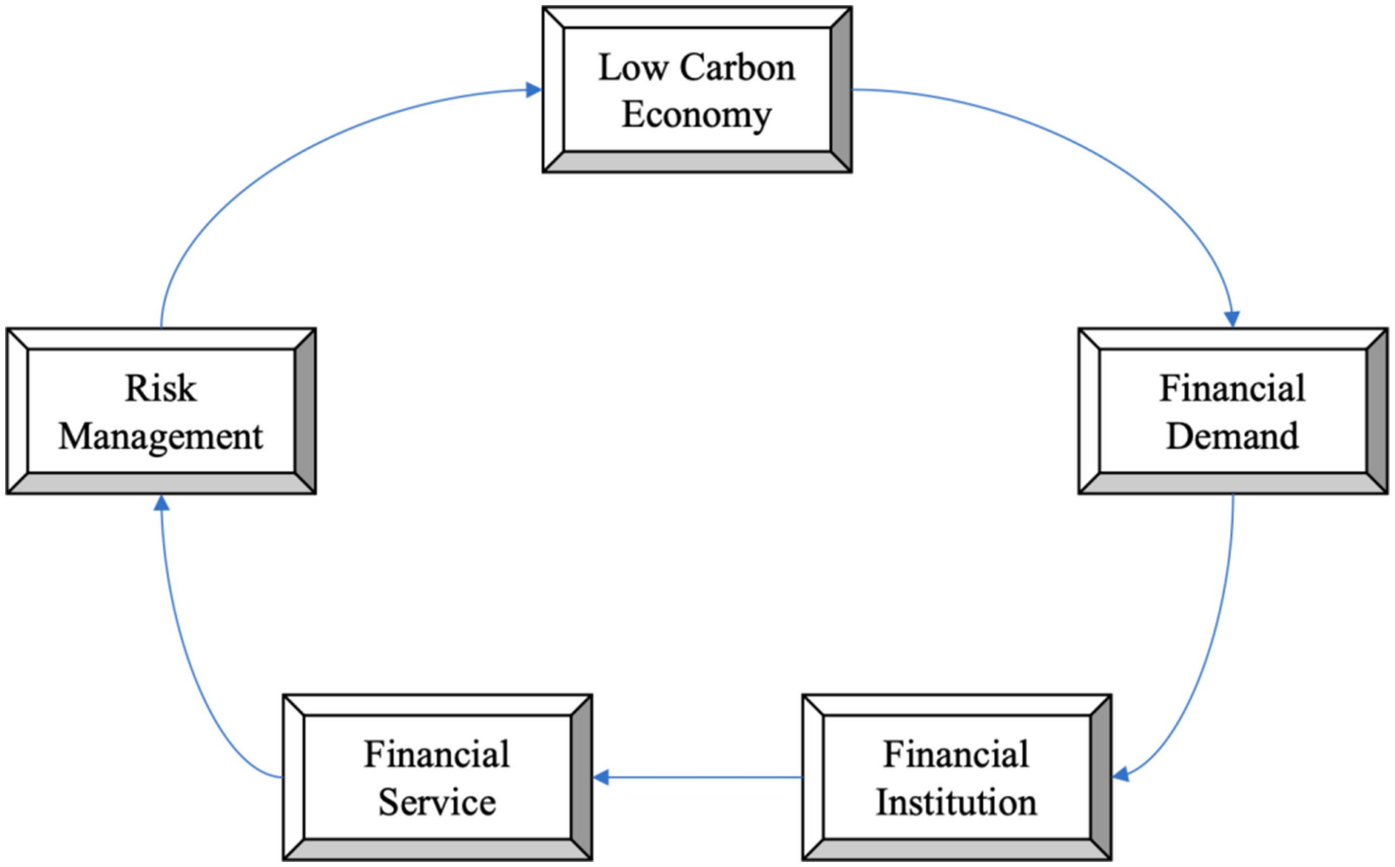
Main pathways for green finance to influence low-carbon economic development.

To bridge the transition to green finance, it is essential to explore how financial mechanisms can support the principles of the green economy. Green finance plays a pivotal role in transforming environmental goals into actionable financial strategies. For example, the judicious and efficient allocation of capital facilitated by green finance can ameliorate issues related to resource mismatches and environmental degradation within the free market, as depicted in Figure 6. This strategic allocation contributes to the development of a low-carbon economy and aligns with the objectives of the green economy.

**Figure 7:**
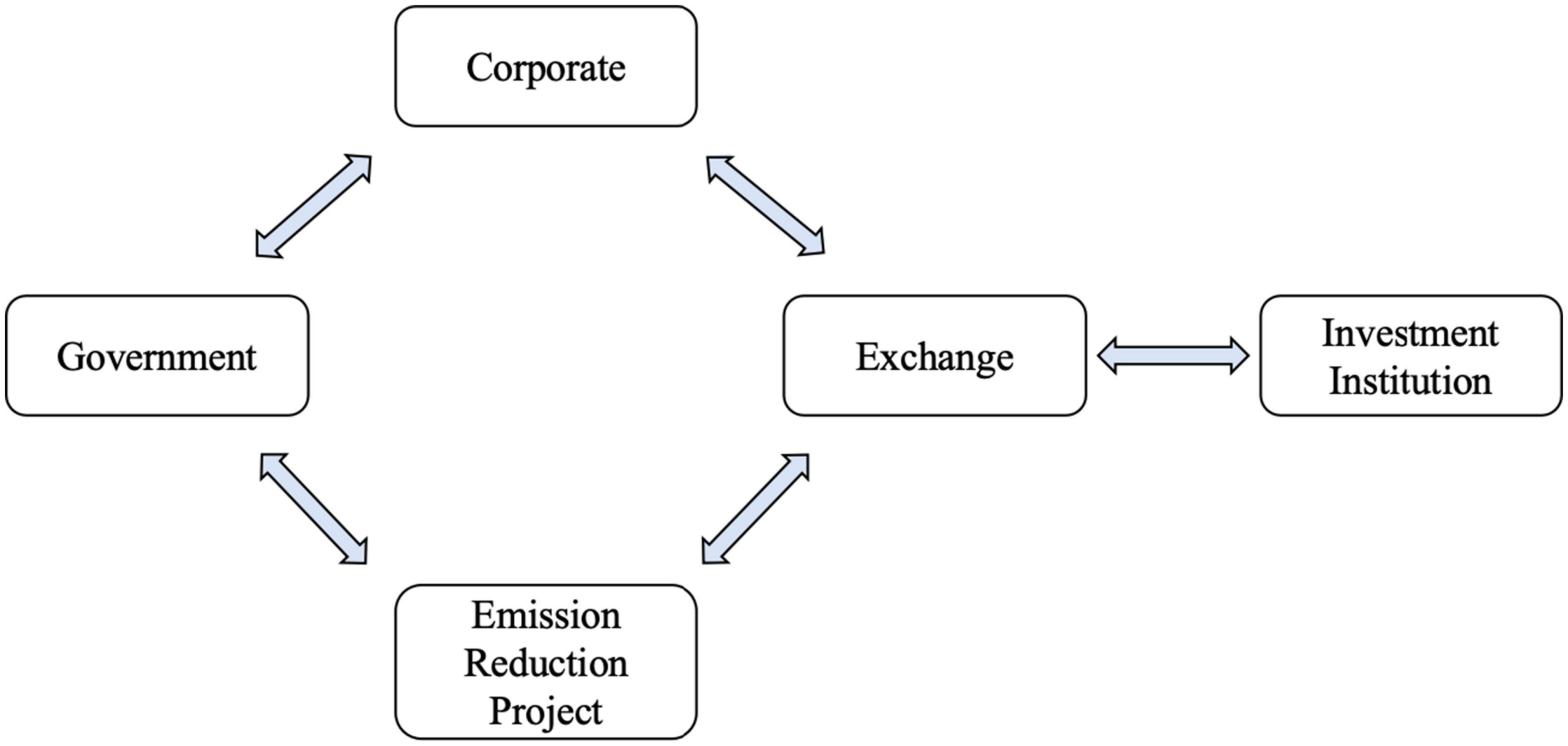
Framework of a carbon finance mechanism.

At present, most resource and technology-intensive enterprises in China have obvious shortcomings in production efficiency and pollution emission control. The reasons for this are low low-carbon awareness and limited technology and production factors. After all, the construction of projects related to low-carbon industries requires a large amount of long-term capital investment, therefore, how to diversify the sources of capital is the primary problem to be solved in promoting the development of low-carbon economy. Carbon finance refers to a range of financing activities for greenhouse gas emission reduction technologies and projects, including carbon trading and bank lending. Carbon finance includes carbon trading by emission credit providers and trading of certified emission reductions by emission credit providers. In addition, market participants can expand financing options through carbon commitments, carbon bonds and carbon funds to support green development and decarbonization by supporting greenhouse gas emission reduction projects in different sectors. Figure 7 illustrates the framework of a carbon finance mechanism.

The core connotation of green development is to establish a regional green economic growth model. Consequently, this requires the region to pay attention not only to the transformation of economic structure in the development process, but also to control the effect of pollution reduction. In other words, to realize the green development of less developed regions, non-resource-consuming industries should be vigorously developed. In addition, it is necessary to promote the green transformation of existing industries, strengthen environmental regulations, reduce pollution emissions, and raise public awareness of environmental protection. In fact, few existing studies have analyzed the digital economy and regional green development in a unified framework. However, there are still many studies that provide research ideas on the role of the digital economy in the green development of the regional economy. The digital economy is key to fostering green development in underdeveloped regions by promoting sustainable economic growth models. It encourages the shift to non-resource-intensive industries like IT, e-commerce, and digital services, which have lower environmental impacts compared to traditional industries. The adoption of digital technologies can enhance efficiency and reduce waste in existing industries, supported by robust environmental policies. Moreover, the digital economy boosts environmental awareness and engagement through educational platforms.

### 2.3 Green economy in less economically developed regions

The concept of green development strives to foster a regional economic growth model characterized by high efficiency, low pollution, and minimal losses. Concurrently, with the accelerated evolution of the green economy, the infusion of information technology and digitalization permeates all facets of economic development, exerting a discernible impact on socio-economic growth mechanisms. The development of the green economy influences economic development through the dual effects of economic growth and industrial structure. Moreover, the green economy contributes to the enhancement of the ecological environment by emphasizing energy conservation and emission reduction. Nevertheless, economic development can potentially compromise environmental quality, establishing a reciprocal relationship wherein environmental quality constrains and, in turn, guides the trajectory of economic development. Consequently, the interplay between economic development and the ecological environment is both mutually constraining and synergistic. Green finance, by influencing economic development and the ecological environment, plays a pivotal role in shaping the trajectory of green economy development.

In less economically developed regions, green economy not only affects economic growth and industrial structure, but also energy conservation and environmental quality. First, under the green economy development model, the government’s financing restrictions on high pollution and high energy consumption enterprises prompt enterprises to change their production patterns. At the same time, the gradual improvement of the information disclosure system enables green finance to obtain and disclose information such as environmental credit and the list of polluting companies in a timely manner. This means that companies will be less likely to violate environmental regulations and will make greater efforts to reduce energy consumption and pollution emissions. What is more, as the concept of green economy becomes more widespread, consumers will consume more and more green products. To some extent, this will help improve consumer behavior. After all, the green economy can encourage consumers to choose energy-efficient and environmentally friendly products and consume in an environmentally friendly way through credit adjustments as well as interest rate subsidies.

## 3 Model construction and research design

### 3.1 Result variable

The selection of energy sources incorporated in the study was predicated on the comprehensive coverage of primary energy types that significantly contribute to carbon dioxide emissions within the Chinese provinces under investigation. The energy sources—raw coal, coke, crude oil, fuel oil, gasoline, kerosene, diesel fuel, and natural gas—were determined based on their prevalence in the energy consumption profiles of the regions and their documented impact on greenhouse gas emissions according to the IPCC guidelines. The rationale for their inclusion lies in their extensive use and the substantial carbon footprint associated with their combustion. These sources represent the major components of the energy mix in the provinces studied, and thus, their inclusion provides a robust basis for the analysis of carbon emissions.

Low carbon means lower emission of greenhouse gasses (mainly carbon dioxide), and lower carbon dioxide emission is used as the result variable. This paper adopts the carbon emission factor method provided by the United Nations Intergovernmental Panel on Climate Change (IPCC) to calculate as shown in Equation 1:


(1) $$LX_{n}=\overset{8}{\underset{y=1}{\sum }}E_{yn}Z_{y}\times \frac{44}{12}$$

Where $LX_{n}$ is the carbon dioxide emission in year *n*; $E_{yn}$ is the consumption of the *y*-th energy source in year *n*; $Z_{y}$ is the carbon emission coefficient of the *y*-th energy source; and *y* is raw coal, coke, crude oil, fuel oil, gasoline, kerosene, diesel fuel, and natural gas.

### 3.2 Conditional variables

The selection of energy intensity as an indicator of technological level in this study was driven by its direct correlation with carbon emissions and its integrative nature, reflecting both the efficiency of energy use and the sophistication of the technological processes in place. Energy intensity, defined as the amount of energy consumed per unit of GDP, serves as a robust indicator of how effectively technology is utilized in the economy to minimize energy consumption while maintaining productivity.

In ensuring a holistic representation of the chosen indicators, the study employs a multi-dimensional strategy: (1) Economic Indicator: *Per capita* GDP has been selected as an indicator of the provinces’ economic status and purchasing power, providing a measure of the overall economic vitality and the potential for investment in cleaner technologies. (2) Demographic Indicators: The total population and urbanization rate are utilized to mirror the demographic challenges and the geographic distribution of inhabitants, essential for gaging energy demand and the feasibility of urban energy conservation efforts. (3) Structural Indicator: The share of the secondary sector within the economy is highlighted to depict the economic structure and identify the main energy-consuming industries that are significant contributors to carbon emissions. (4) Science and Technology Indicator: Energy intensity has been preferred as an indicator of the level of science and technology integration, due to its tangible connection with carbon emissions, thus providing a quantifiable assessment of the environmental impact per unit of economic yield.

**Figure 8:**
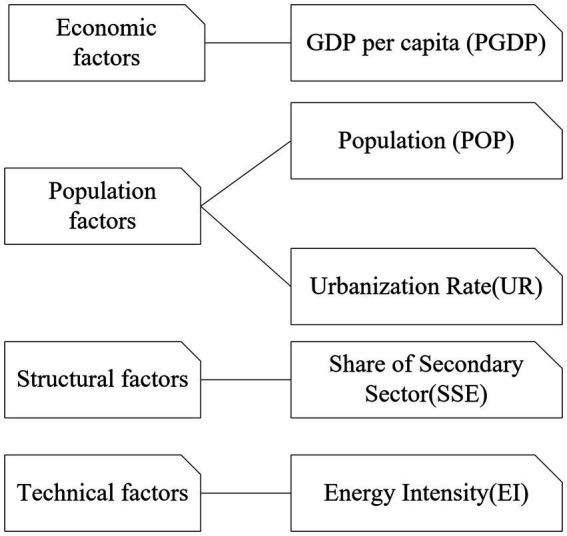
Conditional variables.

Based on the principle of energy balance and the existing literature, this paper integrates a number of factors such as economy, demographics, and science and technology, as shown in Figure 8. Economic factors are measured by GDP *per capita*. Demographic factors are divided into population size and population structure, which are expressed by total population and urbanization rate, respectively. Industrial structure is described by the proportion of secondary industry.

### 3.3 Modeling

**Figure 9:**
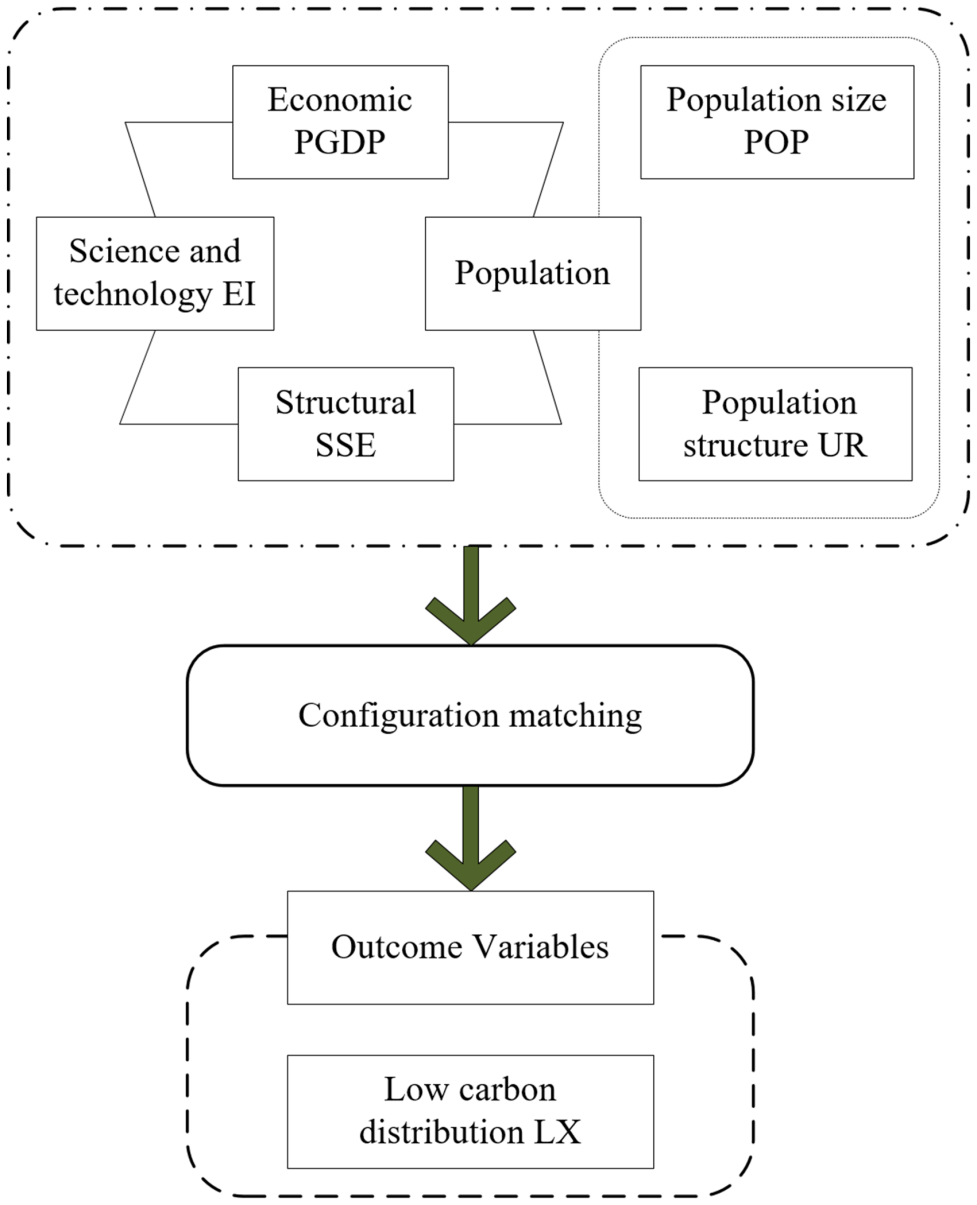
Driving mechanism model of low-carbon development.

Starting from the four key dimensions of economy, population, structure and science and technology, this paper extracts five core factors as independent variables to explore in depth the multiple concurrent factors of low-carbon development and their inherent complex causal relationships. The study aims to answer how these factors interact with each other to effectively reduce carbon emissions. Adopting group thinking, this paper constructs a model of the driving mechanism of low-carbon development based on the conditional and outcome variables, as detailed in Figure 9.

### 3.4 Data calibration

The fsQCA method was chosen for its ability to handle the complexity and nuance inherent in the study of low-carbon development pathways, particularly given the diverse and nuanced causality in social and economic phenomena. fsQCA allows for a more refined analysis by incorporating degrees of membership in conditions and outcomes, rather than the binary presence or absence of conditions as in crisp-set QCA. This nuanced approach is critical for capturing the subtle differences across regions with varying economic profiles and policy implementations. Additionally, fsQCA is particularly adept at dealing with equifinality, the phenomenon where multiple combinations of conditions can lead to the same outcome, which is common in regional development studies. This method enables us to identify multiple pathways to low-carbon development, reflecting the diverse realities of the regions under study.

Taking into account the literature and the actual meaning of the variables, the cut-off points of the variables were set as the upper quartile, median and lower quartile of the sample data. The raw data were transformed into fuzzy set affiliation scores belonging to the range 0–1, as shown in Table 1.

**Table 1:** Calibration of variables.

Variable	Anchor point		
	Full membership	Intersection	Partial membership
LX	59772.68	31672.35	21428.13
PGDP	7.25	4.73	3.87
POP	6364.06	3855.26	2495.56
UR	65.57	57.44	52.15
SSE	48.49	45.26	39.58
EI	0.92	0.57	0.45

In Table 1, “LX” denotes the carbon dioxide emissions, a key measure of environmental impact. “PGDP” represents the *per capita* Gross Domestic Product, indicating the economic status and potential for clean technology investment. “POP” stands for the total population, reflecting demographic challenges and energy demand. The “UR” signifies the urbanization rate, crucial for understanding urban energy conservation. “SSE” is the share of the secondary sector in the economy, highlighting major industries contributing to emissions. Lastly, “EI” is the energy intensity, showing the efficiency of energy use and technological advancement per unit of economic output. These variables were meticulously chosen to encompass economic, demographic, industrial, and technological aspects influencing low-carbon development in China’s less developed regions.

### 3.5 Necessity analysis of individual conditions

Necessary conditions are reflected by consistency. If the consistency is more than 0.9, it means that the conditional variable is a necessary condition for the outcome variable. Five conditional variables’ consistency is less than 0.9, the independence explanation of the individual variables for the low carbon outcome is weak, and there is no necessary condition, as shown in Table 2.

**Table 2:** Analysis of necessary conditions.

Variable	Consistency	Variable	Consistency
PGDP	0.58	~PGDP	0.65
POP	0.42	~POP	0.74
UR	0.55	~UR	0.62
SSE	0.56	~SSE	0.63
EI	0.54	~EI	0.66

“~ “means “not” in logical operations. The definitions of the variables (PGDP, POP, UR, SSE, EI) are consistent with those in Table 1.

### 3.6 Sufficiency analysis of combinations of conditions

A condition combination that leads to the result when it appears is a sufficient condition for the result. The intermediate solution of the result output is the optimized solution, which is more prominent in revelation and universality, as shown in Table 3. The intermediate solution holds significant importance for the universal applicability of the findings, as it encapsulates a balance between the case-specific details and the broader generalizability of the outcomes. Focusing on this intermediate solution facilitates the identification of the pivotal condition combinations that exhibit relevance across the diverse regions examined. Consequently, this enhances the potential for applying the findings to other contexts that share similar characteristics, thereby expanding the scope of the research’s influence and relevance. The results of the complex solution and the intermediate solution in this paper are basically consistent, which indicates that the intermediate solution can adequately reflect the real-life case combinations, and needs to be focused on analysis. The overall coverage of the four condition combinations of the intermediate solution is 0. 55, which can explain about 55% of the cases, and the overall consistency is also high at 0. 92, so these four configurations are sufficient condition combinations to contribute to the low-carbon results.

**Table 3:** Low-carbon pathway configurations.

Conditional variable	Traditional early low carbon path (W1)	Semi-modernized medium-term low carbon path (W2)	Late modernization low carbon path (W3a)	Late modernization low carbon path (W3b)
PGDP	×	×	◎	▲
POP	×	▲	×	×
UR	×	×	◆	◆
SSE	◆	×	×	◎
EI	▲	×	×	×
Typical province	Qinghai	Hunan	Beijing, Shanghai, Tianjin, Hainan	Beijing, Shanghai, Tianjin, Chongqing
Consistency	0.88	0.94	0.96	0.93
Original coverage	0.09	0.13	0.26	0.09
Unique coverage	0.16	0.16	0.03	0.35
Overall consistency	0.93			
Overall coverage	0.56			

◆ indicates that the condition variable appears, and × indicates that the condition variable does not appear; ◎ indicates that the conditional variable can either appear or not appear. The typical provinces (Qinghai, Hunan, Beijing, Shanghai, Tianjin, Hainan, and Chongqing) in the table are the cases corresponding to each of the four different intermediate solutions, which are derived from the output of fsQCA. The definitions of the variables (PGDP, POP, UR, SSE, EI) are consistent with those in Table 1.

To improve the generality of the findings, the following steps were taken in this study.

1. Representative Sampling: A number of provincial administrative districts were selected to ensure that they were representative of the diverse economic and environmental conditions across China.
2. Theoretical Framework: The study’s theoretical framework is based on established theories of green development and externality, ensuring the findings are widely applicable to diverse socio-economic contexts.
3. Policy Implications: The presentation of findings discusses policy implications that transcend the specifics of the sampled regions, concentrating on principles and strategies versatile enough for adaptation to other regions with comparable developmental attributes.

## 4 Low carbon development path analysis

If the antecedent conditions appear in both the parsimonious solution and the intermediate solution, they are the core conditions, which have an important influence on the results. If they only appear in the intermediate solution, they are secondary conditions and play an auxiliary role. The four antecedent configurations were grouped into three paths based on the condition combination characteristics, namely the traditional early low carbon path, the semi-modernized medium-term low carbon path, and the late modernization low carbon path.

### 4.1 Traditional early low carbon path

Path 1: Traditional early low carbon path (referred to as the traditional path or W1). The three core elements of economy, population size and industrial structure are sufficient conditions to promote a low-carbon economy. This path believes that the rapid economic development should not be pursued blindly, but should be kept in a reasonable range, and the economic growth rate should be stabilized, as shown in Table 4. In terms of population, the drastic changes in population size and structure should be reduced, keeping the total population of the provincial government not more than 38.562 million, and slowing down the process of urbanization, with the rate of urbanization not higher than 57.4%. Focusing on the development of the secondary industry, increasing capital investment, raising the share of output to over 45.2%. Since the development of science and technology is at a low level, even if the level of science and technology is improved, it is difficult to reduce carbon emissions in the short term, and the status of science and technology needs to be weakened.

**Table 4:** Characteristics of adjustment targets and paths of conditional variables.

Conditional variable	Traditional early low carbon path (W1)	Semi-modernized medium-term low carbon path (W2)	Late modernization low carbon path (W3)
PGDP	<4.65	<4.65	/
POP	<3856.2	<3856.2	<3856.2
UR	<57.4%	<57.4%	<57.4%
SSE	>45.2%	>45.2%	/
EI	>0.57	<0.57	<0.57
Low carbon initiative	Stabilize the economy, control the population, slow down urbanization, clean up the industry, and weaken science and technology	Stabilize the economy, expand the population, slow down urbanization, accelerate transformation, and strengthen science and technology	Improving quality, accelerating urbanization, strengthening science and technology
Path characteristics	Small economy, low level of science and technology, low urbanization	Upgrading science and technology, rationalization of industrial structure, restricted economic development	Stable population size, high degree of urbanization and technological advancement, which allows for the harmonious development of the economy and the environment

The definitions of the variables (PGDP, POP, UR, SSE, EI) are consistent with those in Table 1.

### 4.2 Semi-modernized medium-term low carbon path

Path 2: Semi-modernized medium-term low carbon path (referred to as semi-modernized path or W2). Population structure, industrial structure and science and technology level are the core conditions, constituting a combination of sufficient conditions for low-carbon development. In order to create a clean and low-carbon production and living space, it is necessary to take into full consideration the negative impacts of rapid urbanization, slow down the urbanization process in an orderly manner, and reduce the urbanization rate to below 57.3%. At the same time, promote the integrated development of multiple industries, with the proportion of the secondary industry below 45.4%, and build an industrial system in harmony with the population, resources and environment. Encourage scientific and technological innovation, increase investment in technology and capital, develop renewable energy, and strive to reduce energy intensity to 0.57. In addition, this path also includes two secondary conditions, namely, population and smooth economic development, and gives full play to the role of human beings in reducing carbon emissions and focusing on the high-quality development of the economy.

### 4.3 Late modernization low carbon path

Path 3: Late modernization low carbon path (modernization Path or W3). The combination of population size, demographic structure, and technological level is a sufficient condition to promote low-carbon development. The government should promote urbanization, increasing the urbanization rate to over 57.4%. At the same time, it should control the population size so that the provincial population is less than 38.562 million. Improve the quality of population, emphasize scientific and technological innovation, and reduce the energy intensity to below 0. 57, and explore the efficient low-carbon development path. Specifically, Path W3a should adjust the industrial structure, accelerate industrial transformation and upgrading, upgrade the level of industries, and establish a modern industrial system based on the service industry, in addition to the elements of population and science and technology. Path W3b emphasizes stimulating the vitality of economic development, comprehensively raising *per capita* GDP, and maintaining sustained and healthy economic development. This path indicates that economic development and the construction of a low-carbon society do not have to be opposed to each other, and that a win-win situation for environmental protection and economic development can be realized as long as other conditions are well coordinated.

A comparison of the three paths shows that W1 reduces carbon by means of multifaceted restrictions on economy, population, urbanization and science and technology, and advocates non-development and non-pollution. The W2 strategy is to further relax population control on the basis of W1, and to promote emission reduction and carbon reduction by attracting talents. It achieves more advanced low-carbon development by accelerating industrial upgrading and reducing the proportion of secondary industries, while incentivizing scientific and technological innovation and technological upgrading. W3 further liberalizes restrictions on the economy and industrial structure, upgrading the quality of the population and upgrading the level of the industry, which is the most desirable low-carbon path. On the whole, the population shifts from quantitative growth to quality improvement, urbanization level increases, industrial layout is reasonable, scientific and technological innovation is important, economy and environment are coordinated, and W1 to W3 are gradually optimized.

### 4.4 Challenges and obstacles

The journey toward low-carbon development is fraught with multifaceted obstacles, spanning political, economic, and social domains. These challenges, while substantial, are not insurmountable with targeted and strategic measures. Below, we delve into each category, linking the challenges to our earlier findings and discussing their specific implications for the transition toward a low-carbon economy in less developed regions.

#### 4.4.1 Political challenges

Political challenges, notably policy uncertainty and inconsistent enforcement, can significantly hinder the low-carbon development, particularly for regions following the traditional early low-carbon path where a stable policy environment is essential. To address these, we recommend enhancing interdepartmental coordination to ensure policy consistency and stability. Establishing a consultative platform with multiple stakeholders can promote transparency and public participation in the policy-making process, thus fostering an environment conducive to long-term strategic planning and policy adherence.

#### 4.4.2 Economic challenges

Economic challenges, such as financial constraints and insufficient investment in low-carbon technologies, are pronounced in the semi-modernized mid-carbon path regions, which are striving to balance industrial upgrading with economic growth. To overcome these, fiscal incentives like tax relief, subsidies, and low-interest loans can encourage private sector investment. Leveraging green finance mechanisms to direct funding toward low-carbon projects is also crucial, as it not only fosters investment but also aligns financial flows with sustainable development goals.

#### 4.4.3 Social challenges

Social barriers, including low public awareness and acceptance of low-carbon development, are universal but have distinct implications for the post-modernized low-carbon path regions, where urbanization and education levels are higher. These regions have the potential to lead by example in adopting sustainable practices, yet they require broad societal support to realize this potential. Therefore, we suggest launching education and awareness campaigns to enhance understanding of low-carbon lifestyles and initiating community engagement programs to promote public participation and support for low-carbon initiatives.

By connecting these challenges to the specific conditions and characteristics of each development path, we provide a clearer picture of how they might impede or facilitate the low-carbon transition. This understanding is vital for devising targeted strategies that can effectively navigate these obstacles and accelerate the shift toward a sustainable, low-carbon future for less economically developed regions.

### 4.5 Policy recommendations and government interventions

To ensure the effective implementation of low-carbon development pathways and to overcome the aforementioned political, economic, and social challenges, a series of targeted policy measures and government interventions are required. These measures aim to facilitate the coordination of economic growth with environmental protection and ensure the successful implementation of low-carbon development pathways.

#### 4.5.1 Policy measures

Enhanced Interdepartmental Coordination: Establish a high-level interdepartmental coordination mechanism to ensure policy consistency and synergies across different government departments, reducing policy uncertainty and enforcement disparities.

Fiscal Incentives: The government should offer fiscal incentives such as tax exemptions, subsidies, and low-interest loans to encourage businesses and individuals to invest in low-carbon technologies and projects.

Green Finance: Develop the green finance market by leveraging green bonds, green funds, and other financial instruments to mobilize private capital for low-carbon projects.

Education and Awareness: Conduct public education and awareness campaigns to increase understanding and acceptance of low-carbon lifestyles.

#### 4.5.2 Government interventions

Regulations and Standards: Formulate and enforce stringent environmental regulations and standards to limit pollutant emissions and encourage businesses to adopt more eco-friendly production methods.

Public Investment: Increase public investment in low-carbon technology research and infrastructure development to reduce the market cost of these technologies and promote their widespread adoption.

Public-Private Partnerships (PPP): Encourage collaboration between the government and private sector through Public-Private Partnership models to implement low-carbon projects.

Community Engagement: Support community involvement in low-carbon development projects to ensure they meet local needs and enhance the social acceptance of such initiatives.

## 5 Discussion

Our comparative analysis indicates that the effectiveness of low-carbon development strategies is highly dependent on the regional context. Therefore, we have identified several regions with distinct characteristics to illustrate how specific features should influence strategy adoption:

Qinghai Province: As a less economically developed region with abundant clean energy resources, Qinghai should focus on strategies that capitalize on its renewable energy potential. This includes investing in solar and wind energy infrastructure to promote a clean energy economy.

Hunan Province: With a strong manufacturing base and a higher level of economic development, Hunan’s strategy should emphasize industrial upgrading and the adoption of energy-efficient technologies to reduce its carbon footprint while maintaining economic growth.

Beijing, Shanghai, Tianjin, and Chongqing Municipalities: These urban centers, characterized by high population density and advanced service sectors, should prioritize green urban planning and public transportation systems to reduce carbon emissions from transportation and urban sprawl.

Hainan Province: With its unique ecological environment and a focus on becoming an international tourism destination, Hainan should adopt strategies that protect its natural resources and promote sustainable tourism practices.

These instances illustrate how various regions, contingent on their developmental stages and characteristics, can implement customized low-carbon development strategies to ensure equilibrium in economic, social, and environmental advancement.

## 6 Conclusion

The study provides a thorough analysis of green and low-carbon development pathways in China’s less economically developed regions. It identifies and evaluates three distinct pathways: the traditional early low-carbon path, the semi-modernized mid-carbon path, and the post-modernized low-carbon path. Each pathway is tailored to the specific developmental stage and characteristics of the region, emphasizing a balanced approach to economic growth and environmental sustainability. The research highlights the critical role of green finance and the establishment of a robust green fiscal policy system in facilitating the transition to a low-carbon economy. The findings indicate that adopting suitable low-carbon development strategies can enhance environmental innovation capacity and global competitiveness, thereby contributing significantly to China’s overall economic development. Specifically, the traditional early low-carbon path focuses on stabilizing economic growth and controlling population size while advancing the clean energy sector. The semi-modernized mid-carbon path builds on this foundation by attracting talent, accelerating industrial upgrading, and fostering scientific and technological innovation. The post-modernized low-carbon path further relaxes restrictions on economic and industrial structures, emphasizing improvements in population quality and technological innovation to achieve a balance between economic development and environmental protection.

This study offers key insights into low-carbon development strategies for less economically developed regions in China but recognizes its limitations. The research is confined to a select group of provincial regions, limiting the generalizability of its findings. Additionally, the conclusions are based on a current snapshot, and the long-term viability of the proposed strategies is yet to be confirmed. Future research should broaden the study’s scope to encompass a wider range of regions and extend the time frame for analysis. Incorporating dynamic and real-time data could enhance the accuracy of assessing low-carbon development trajectories. It is also suggested that further studies delve into the mechanisms and policies that can foster a sustainable transition to a green and low-carbon economy, considering the impact of technological innovation, market incentives, and government policies. In addition, future studies will be conducted to monitor the long-term effectiveness of the proposed strategy, including a combination of dynamic and real-time data.

## Data Availability

The original contributions presented in the study are included in the article/supplementary material, further inquiries can be directed to the corresponding author.
